# An Assay Using Localized Surface Plasmon Resonance and Gold Nanorods Functionalized with Aptamers to Sense the Cytochrome-*c* Released from Apoptotic Cancer Cells for Anti-Cancer Drug Effect Determination

**DOI:** 10.3390/mi8110338

**Published:** 2017-11-22

**Authors:** Jacky Fong-Chuen Loo, Pui-Man Lau, Siu-Kai Kong, Ho-Pui Ho

**Affiliations:** 1 Department of Biomedical Engineering, The Chinese University of Hong Kong, Shatin, New Territories, Hong Kong, China; jackyfcloo@gmail.com; 2 School of Life Sciences, The Chinese University of Hong Kong, Shatin, New Territories, Hong Kong, China; irenelau@cuhk.edu.hk (P.-M.L.); skkong@cuhk.edu.hk (S.-K.K.)

**Keywords:** aptamer, gold-nanorod, localized surface plasmon resonance

## Abstract

To determine the degree of cancer cell killing after treatment with chemotherapeutic drugs, we have developed a sensitive platform using localized surface plasmon resonance (LSPR) and aptamers to detect the extracellular cytochrome-*c* (cyto-*c*), a mitochondrial protein released from cancer cells for the induction of apoptosis after treatment, to evaluate the effectiveness of cancer therapy. In this assay, a short single-stranded 76-mer DNA aptamer with a unique DNA sequence, which binds towards the cyto-*c* like an antibody with a high binding affinity and specificity, was conjugated to gold nanorods (AuNR) for LSPR sensing. Practically, cyto-*c* was first grabbed by a capturing antibody functionalized on the surface of micro-magnetic particles (MMPs). Subsequently, the AuNR-conjugated aptamer was added to form a complex sandwich structure with cyto-*c* (i.e., (MMP-Ab)-(cyto-*c*)-(AuNR-aptamer)) after washing away the non-target impurities, such as serum residues and intracellular contents, in a microfluidic chip. The sandwich complex led to formation of AuNR aggregates, which changed the LSPR signals in relation to the amount of cyto-*c*. With the LSPR signal enhancement effects from the AuNRs, the detection limit of cyto-*c*, sparked in human serum or culture medium, was found to be 0.1 ng/mL in our platform and the whole sensing process could be completed within two hours. Moreover, we have applied this assay to monitor the apoptosis in leukemia cancer cells induced by a potential anti-cancer agent phenylarsine oxide.

## 1. Introduction

Cytochrome-*c* (Cyto-*c*) is a potential marker for diagnosing many diseases, such as myocardial infarction, and determining the effectiveness of cancer therapy [1,2]. During the process of programmed cell death (apoptosis) and/or necrosis, cyto-*c* is released rapidly in minutes from the mitochondria to the cytosol and to the extracellular environment in both in vitro and in vivo conditions [3,4]. Serum cyto-*c* concentrations have been reported as a good indicator to predict the survival rate of patients after resuscitation from cardiac arrest; to evaluate the conditions of trauma–hemorrhage induced hypoxia and organ dysfunction; to monitor the aging heart damage in healthy elderly; to serve as the danger-associated molecular patterns (DAMP); to assess the severity of organ damage; and to predict the prognosis in the patients with systemic inflammatory response syndrome/multiple organ dysfunction syndrome (SIRS/MODS) [5,6,7,8]. In all these examples, non-survivors were usually found with a high concentrations of cyto-*c* in their plasma. In cancer therapy, apoptosis is the primary process that eliminates cancer cells after drug treatment and the release of cyto-*c* from the mitochondria into the cytoplasm is an essential step and is regarded as a ‘point-of-no-return’ stage of apoptosis. Indeed, the serum level of cyto-*c* increased at the onset of chemotherapy, reached the peak with a median of two days, and decreased gradually during remission induction [9,10]. Therefore, the concentration of cyto-*c* found in the blood circulation correlates to the percentage of cancer cell death, i.e., the efficacy of the anti-cancer drug treatment [11,12].

Previously, we have developed a number of aptamer-based bio-barcode (ABC) assays, in which a 76-mer DNA aptamer was employed to capture cyto-*c* selectively, and it also acts as both the recognition element and the bio-barcode reporter in the ABC assays [13,14]. This aptamer is a small single-chain DNA with a unique nucleotide sequence, which self-folds into a unique three-dimensional shape to bind cyto-*c* with high affinity and high specificity in a way similar to that in the antibody-antigen reactions. Due to its unique DNA sequence, it can serve as a simple bio-barcode reporter in the ABC system for the sandwich complexes ((micro-magnetic particles (MMP)-capturing antibody (Ab))-target-(aptamer)) after DNA amplification. These ABC assays have been introduced previously to sensitively and rapidly screen the cell death marker in multidrug resistant cancers as part of the drug screening process [12,15]. In these ABC assays, most of them required a DNA amplification step and an introduction of DNA-labeling dyes to generate fluorescence as a means of signal detection. Yet, this approach takes time and sometimes yields low signal-to-noise ratio readouts [11,16]. Therefore, label-free sensitive methods without DNA amplification using simple operational steps have been proposed as the next-generation biosensing technique.

Nanomaterial-based localized surface plasmon resonance (LSPR), which employs a disconnected pattern of the metallic surface on nanomaterials to generate a non-propagating plasmon, has been drawing much attention these days because the LSPR platform provides several advantages over other optical label-free techniques. First, different functionalized nanomaterials coated with recognition elements can be applied in a solid or a solution format for high-throughput target detection [17]. Second, the signal readout, as an obvious color change, makes the robust absorbance scanning detection by spectrophotometer become feasible [18]. Third, LSPR is insensitive to temperature change and, therefore, temperature stabilization is not needed for sensitive signal acquisition [19]. More importantly, solution-phase LSPR sensors using aqueous functionalized nanoparticles (NP), instead of surface immobilization, simplifies the detection process, which only involves simple mixing of the functionalized NP and the sample of interest in an aqueous environment. Since the sensing of LSPR signals relies on the change in the refractive index, spectrum scanning is commonly used to measure the LSPR spectrum, as well as the absorbance at a particular wavelength, usually at the peak region [17,18]. Among different NPs, gold nanorods (AuNRs) have demonstrated excellent characteristics as the core element in the LSPR biosensors, including a higher sensitivity of the refractive index and a longitudinal plasmon systematically tunable by simply adjusting their size and aspect ratio [20,21,22]. Moreover, the NRs have showed a higher LSPR sensitivity compared with that of nanospheres [23,24,25].

**Figure 1:**
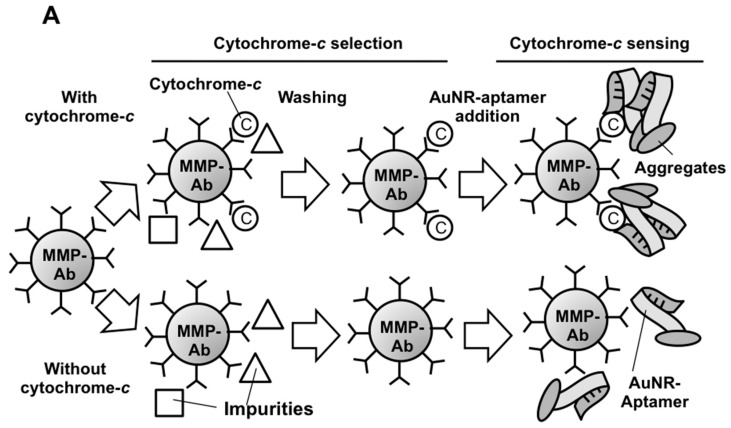
Gold nanorod localized surface plasmon resonance (AuNR-LSPR) assay for cyto-*c* sensing. A schematic diagram showing the procedures in our AuNR-LSPR assay. Cytochrome-*c* (Cyto-*c*) molecules in the serum were firstly grabbed specifically by cyto-*c* capturing Abs on functionalized micro-magnetic particle (MMP) surface. After washing under a magnetic field, AuNR-functionalized DNA aptamers against cyto-*c* were added to generate ((MMP-Ab)-(cyto-*c*)-(AuNR-aptamer)) sandwich complexes. The change in the absorbance spectrum due to the LSPR effect was recorded.

On the other hand, microfluidics adds another dimension to enhance the robustness of detection. Microfluidic chips provide a micron-scale fluidic actuation with advantages over the current benchtop method by reducing the volume and the size of liquid handling. Additionally, sequential fluidic flow of the assay can be controlled by fluidic actuation of different channels of reagents (Figure 1). Therefore, we report here for the first time the utilization of this LSPR-AuNR assay for rapid cyto-*c* sensing in human serum on a microfluidic platform using a label-free LSPR approach without DNA amplification. As shown in Figure 1, the aptamer immobilized on an AuNR as the functionalized unit was used to detect the cyto-*c* bound by the capturing Ab on MMP. Mimicking the clinical situations during chemotherapy, cyto-*c* of the indicated concentrations were sparked in human serum. Results from our study confirmed that this LSPR-AuNR assay is a convenient and effective approach for determining the concentration of cyto-*c* in the serum. With the growing demand on the healthcare market, especially the need of a convenient, user-friendly, and sensitive point-of-care (POC) biosensor for bedside utility, it is likely that our assay in the microfluidic platform will go beyond academic communities to be a practical POC biosensor for clinical diagnosis and prognosis.

## 2. Materials and Methods 

### 2.1. Reagents and Human Serum Samples

Aptamer was synthesized by Integrated DNA Technologies. The sequence of the 76-mer cyto-*c* biotinylated-aptamer was 5′-biotin-TEG-*ATCGATAAGCTTCCAGAGCC*GTGTCTGGGGCCGACCGGCGCATTGGGTACGTTGTTGCCG*TAGAATTCCTGCAGCC*-3′, where the italics represent the constant region of our aptamer during the systematic evolution of ligands by exponential enrichment (SELEX) process in our previous reports [14,15]. Biotin-TEG, where the TEG (triethylene glycol) is the extended spacer arm of 15 atoms between aptamer and biotin to reduce hindrance of aptamer-target binding, was added at the 5′ region of the aptamer [14]. MMP Protein A magnetic beads, with a mean diameter of 2 μm, nonporous superparamagnetic microparticles (NEB S1425S) were obtained from New England BioLabs. Anti-Cyto-*c* Ab (sc-7159) was obtained from Santa Cruz Bio-technology (Santa Cruz, CA, USA). Recombinant streptavidin was obtained from Life Technologies (Thermo Fisher Scientific, Waltham, MA, USA). Cyto-*c*, bovine serum albumin (BSA), phenylarsine oxide (PAO), and other reagents were purchased from Sigma-Aldrich. Fetal bovine serum and horse serum were obtained from Life Technologies. Human blood was obtained from healthy donors by puncturing fingertips with sterile lancets with depth settings with informed consent and approval by the University Clinical Research Ethics Committee (CRE-2012.025). To obtain the human serum, the non-heparinized blood was centrifuged at 2000× *g* for 10 min and the supernatant was collected. All sera were diluted to 10% (*v*/*v*) with sterile phosphate buffered saline (PBS) before use.

### 2.2. AuNR Preparation and Functionalization

AuNR (length:width = 47.3 ± 2.4:22.3 ± 1.6 nm) were synthesized as described in our previous publication and stabilized with cationic surfactant cethyltrimetylammonium bromide (CTAB) [15,26]. To remove the CTAB for aptamer conjugation, Polyethylene glycol (PEG) displacement was performed using methoxy polyethylene glycol thiol (mPEG-SH) 1000 and thiol-PEG-carboxyl (HS-PEG-COOH) 3400. mPEG-SH and HS-PEG-COOH with different ratios in a total concentration of 1 mM were added on AuNRs (OD_630 nm_ = 0.2) and the mixture was incubated overnight at room temperature. The PEG-displaced AuNRs were washed twice with DNase-free deionized water using centrifugation at 8000× *g* for 10 min. *N*-ethyl-*N*′-(3-(dimethylamino) propyl) carbodiimide/*N*-hydroxy succinimide (EDC/NHS) chemistry was conducted to activate the carboxyl group of HS-PEG-COOH for amine group attachment. Briefly, 0.4 M EDC and 0.15 M (NHS) were prepared freshly and mixed in a 1:1 ratio. The mixture was then loaded to the PEG-displaced AuNRs and incubated for 15 min at room temperature. The ECD/NHS was removed by centrifugation and streptavidin (100 μg/mL) was then added and incubated for 30 min. To visualize AuNR targeting the MMP-Ab-cyto-*c* complex by confocal microscopy (Leica SP8, Leica Microsystems Inc., Wetzlar, Germany), enhanced green fluorescent protein (EGFP) (100 μg/mL) was added with streptavidin during protein binding on the AuNRs’ surface. Blocking agent ethanolamine (10 mM) was added to the mixture for 10 min to block all activated carboxyl groups. Biotinylated-aptamer (10 pmol) was then added and incubated for another 30 min. The AuNR-aptamers were washed twice with DNase-free deionized water and ready for the downstream AuNR-LSPR assay. 

To validate the proper functionalization of AuNR-aptamers, AuNRs from each sequential step of functionalization were mixed with SYPRO protein (Thermo Fisher Scientific, Waltham, MA, USA) dye for protein quantification or SYBR safe (Thermo Fisher Scientific, Waltham, MA, USA) DNA binding dye for DNA quantification in a 96-well plate and incubated for 30 min at room temperature, prior to the fluorescence detection with excitation/emission 485/625 nm and 485/540 nm for protein and DNA measurements, respectively. BSA of standard concentrations from 0 μg/mL to 100 μg/mL was used to calculate the protein conjugated on the AuNRs. Aptamer DNA of standard concentrations from 0 nM to 1000 nM was used to calculate the aptamer conjugated on the AuNRs.

### 2.3. Microfluidics Design and Fabrication 

**Figure 2:**
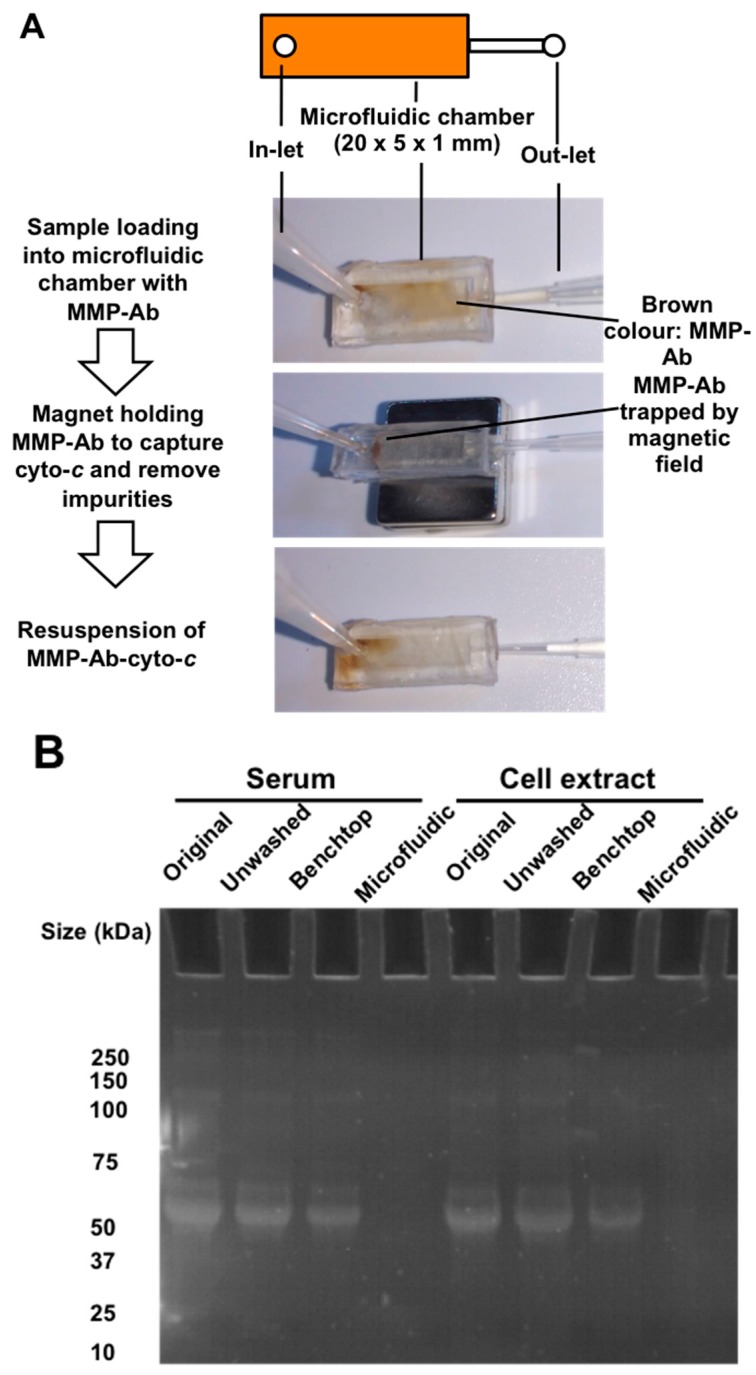
Use of a microfluidic chip to improve impurities removal. A schematic diagram and photos show the microfluidic chip with a reaction chamber in the dimension of 20 × 5 × 1 mm for holding MMPs during the fluidic flow under a fixed magnetic field (**A**). The gel photo shows a comparison of impurities removal between benchtop and microfluidic chip methods during cytochrome-*c* selection in serum and culture medium (**B**).

The disposable microfluidic chamber, a polydimethylsiloxane (PDMS)-based polymer, of length × width × depth of 20 × 5 × 1 mm, was replicated from a poly(methyl methacrylate) (PMMA) mold fabricated with computer numerical control milling. Liquid PDMS was poured onto the PMMA mold and incubated at 60 °C for 2 h. The bottom of the solidified PDMS microfluidic chip was then sealed with a transparent adhesive purchased from Life Technologies. The inlet and outlet were connected with the tip of a micropipette to guide the fluid (Figure 2A).

### 2.4. AuNR-LSPR Assay

The MMP was conjugated with mouse anti-cyto-*c* Ab at 25 °C for 1 h and then blocked with 1% BSA in the binding buffer (10 mM HEPES, 75 mM NaCl, 5 mM MgCl_2_, 1 mM CaCl_2_, pH 7.4) at 25 °C for 1 h. The MMP-Ab was then loaded into the microfluidic chamber with permanent magnet placed at the bottom to hold the MMP-Ab (Figure 2A). Cyto-*c* was prepared as a 5 mg/mL stock solution in DNase-free deionized water and then diluted in PBS buffer, sera, or cell culture Roswell Park Memorial Institute (RPMI)-1640 medium (Thermo Fisher Scientific, Waltham, MA, USA) with 20% (*v*/*v*) fetal bovine serum (FBS). The standard cyto-*c* solutions or samples were loaded into the microfluidic chamber and incubated at 25 °C for 1 h. After that, the MMP were then washed once with 1 mL binding buffer plus 0.1% tween-20 and then with 1 mL DNase-free deionized water at a flow rate of 500 μL/min. Fifty microliters AuNR-aptamer was then added into the microfluidic chamber without magnet holding, and the complex was added into a 96-well plate for downstream detection by scanning the absorbance from 400 nm to 1000 nm with an absorbance reader (Tecan Spark 10M, Tecan Group Ltd., Zürich, Switzerland).

To evaluate the effectiveness of impurities removal by the assay using the microfluidic chip, compared with benchtop method on effectiveness of impurities removal, serum (10% FBS) and cell extract (1 × 10^5^/mL HL60 cell lysate, Santa Cruz Biotechnology, Dallas, TX, USA) samples were used. A benchtop washing method was followed in previous studies, except a one-time washing with 1 mL buffer, i.e., the same volume as that of the microfluidic chip method, for a fair comparison [13]. Protein gel electrophoresis was conducted to profile the presence of impurities from the serum and intracellular content. Briefly, samples, including the original sample, unwashed sample after binding on MMA-Ab, and washed samples from the benchtop method or microfluidic chip, were loaded into the gradient 4–20% polyacrylamide sodium dodecyl sulfate (SDS) protein-denatured gel (Bio-Rad Laboratories, Inc., Hercules, CA, USA) after 10-min boiling with SDS loading dye. The electrophoresis was set at 140 V for 40 min. The gel was then stirred with protein binding fluorescence 1X SYPRO red (Sigma-Aldrich Corporation, St. Louis, MO, USA) dye in 7.5% acetic acid for 30 min before ultraviolet (UV) illumination as shown in Figure 2B. Protein bands appeared in the original samples and samples from the unwashed and benchtop method washing, while no bands appeared in either serum or cell extract samples from the microfluidic chip washing method on both serum and cell extract samples, indicating the high effectiveness of microfluidic chip for impurities removal (Figure 2B).

### 2.5. Cancer Cells and Anti-Cancer Drug Treatment

Human acute promyelocytic leukemia HL-60 cells were obtained from ATCC (CCL-240, American Type Culture Collection, Manassas, VA, USA). For studying the anti-cancer effect of PAO, HL-60 cells cultured in the RPMI medium with 20% (*v*/*v*) FBS were collected, washed twice with PBS, and resuspended to 1 × 10^5^/mL in serum and treated with PAO of various concentrations or positive control (digitonin (5 μM)) were loaded and incubated at 37 °C for 1 h. Cell-free serum was collected by passing the sample through a 0.2 μm filter unit. The serum was then subjected to AuNR-LSPR assay. The cells were also subjected to Annexin-V-GFP (BioVision Inc., Milpitas, CA, USA) and Propidium iodide (PI) staining for flow cytometric analysis to determine the early and late apoptotic response. Briefly, the cells treated with PAO for one hour were stained with Annexin-V-GFP (final concentration: 50 μg/mL) for 10 min and PI (final concentration: 2.5 μg/mL) for 20 min in the dark. The cells were then washed twice with PBS. Subsequently, green and red fluorescence were detected with an excitation at 488 nm by a flow cytometer (BD FACSVerse, Becton, Dickinson and Company (BD), Franklin Lakes, NJ, USA). The fluorescence properties of 1000 cells were collected for the analysis.

### 2.6. Data Analysis

The LSPR signals were processed with the software GraphPad Prism (version 5.0, GraphPad Software, Inc., La Jolla, CA, USA). Results are mean ± standard error of the mean (SEM) from at least three independent assays. Statistical analysis of the experimental results was conducted by Student’s *t*-test, and *p*-values less than 0.05 were considered statistically significant.

## 3. Results and Discussion

### 3.1. Optimization of AuNR-Functionalized Aptamer

**Figure 3:**
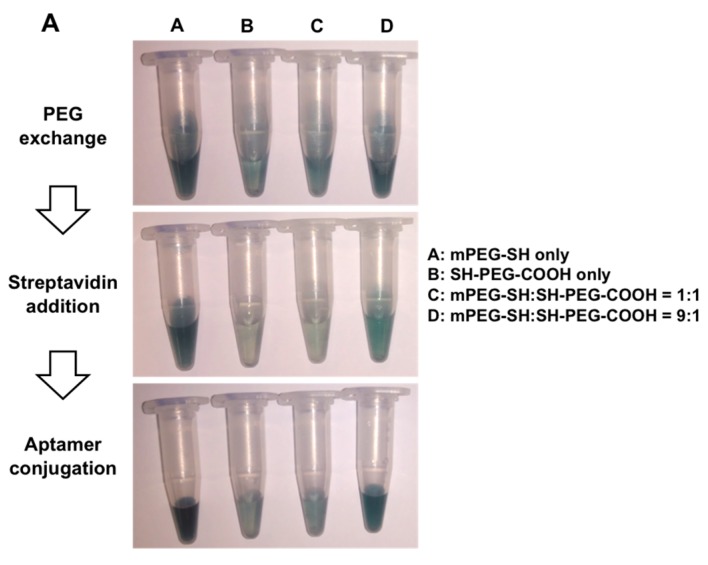

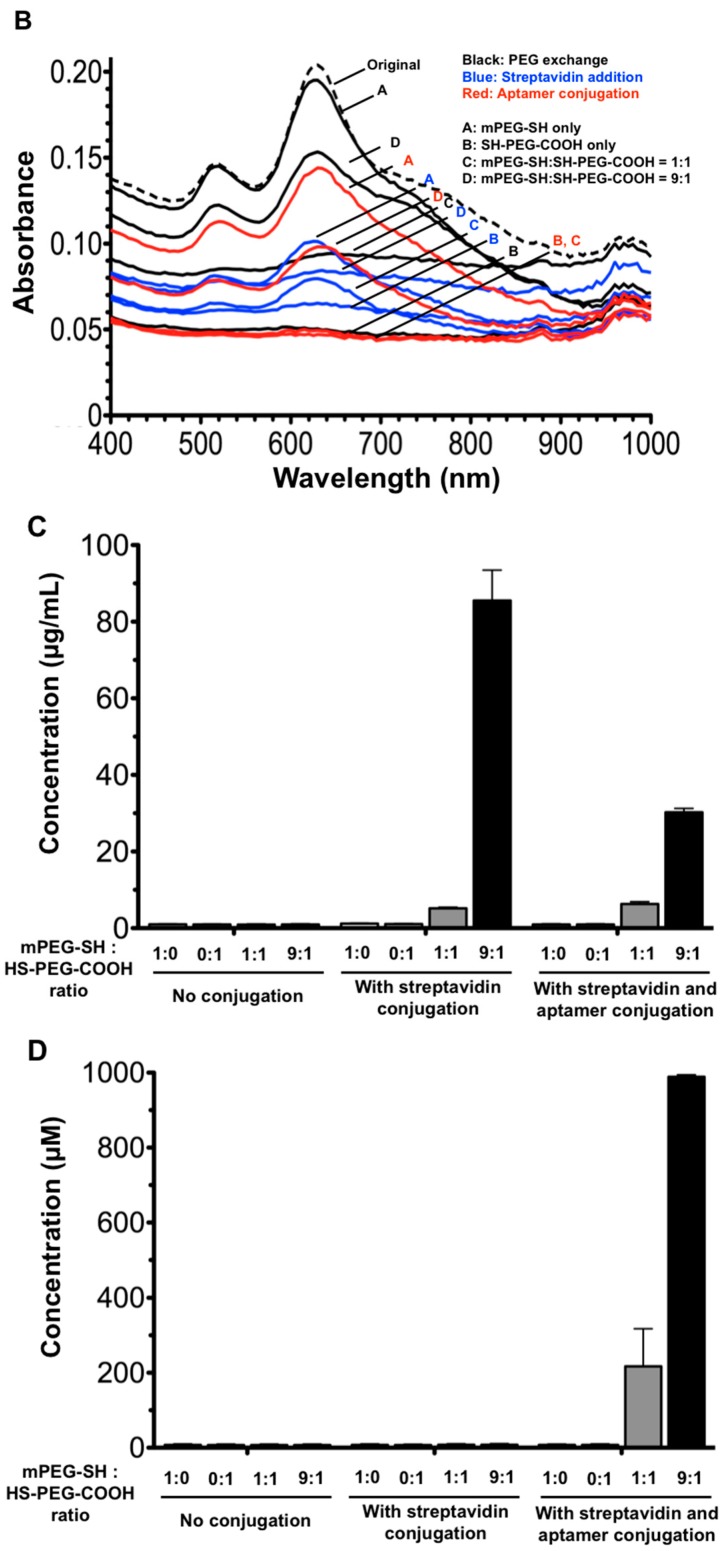
AuNR functionalization. Photographs (**A**) and absorbance spectra (**B**) of AuNRs upon PEG displacement, streptavidin addition, and aptamer conjugation during surface modification with different ratios of mPEG-SH:HS-PEG-COOH. The blue color represents the non-precipitated, suspended AuNR, while the colorless change indicates that AuNR precipitation has occurred. The bar chart shows the evaluation of streptavidin (**C**) and aptamer (**D**) binding during AuNR functionalization. Fluorescence SYPRO red dye was used to evaluate the amount of streptavidin protein bound, and DNA binding fluorescence SYBR safe dye was used to evaluate the amount of bound aptamer DNA.

Prior to the cyto-*c* sensing application, the optimization of aptamer conjugation on AuNRs was conducted to obtain the best functional nano-sensing units. PEG with sulphyl (-SH) group only (mPEG-SH), and PEG with both a sulfur group and carboxyl (-COOH) group (HS-PEG-COOH) were used to displace CTAB from AuNRs. Since only the latter provides the carboxyl group for further AuNR functionalization, the ratio of mPEG-SH to HS-PEG-COOH was adjusted to optimize the condition for AuNR functionalization without losing its stability due to CTAB removal. Figure 3A shows the photographs of AuNRs upon PEG displacement, streptavidin and aptamer addition during surface modification with different mPEG-SH:HS-PEG-COOH ratios. The blue color represented the non-precipitated, suspended AuNRs, while a colorless change indicated that precipitation of AuNRs had occurred. It could be observed that the PEG coating with mPEG-SH alone or mPEG-SH:HS-PEG-COOH in a 9:1 ratio led to AuNRs in suspension form, while the PEG coating with HS-PEG-COOH and mPEG-SH:HS-PEG-COOH in a 1:1 ratio produced the precipitation of AuNRs, as evidenced by the decolorizing of the blue color. Absorbance spectrum of AuNRs upon PEG displacement, and streptavidin and aptamer addition was also scanned (Figure 3B). First, the overall decrease in the absorbance spectrum upon functionalization procedures indicated there was a loss in the AuNRs during centrifugation and resuspension. Second, a larger decrease in the longitudinal surface plasmon band was observed, i.e., the absorbance peak at 630 nm, in the preparation using PEG coating with HS-PEG-COOH only or mPEG-SH:HS-PEG-COOH of 1:1 ratio was observed, which was in agreement with Figure 3A, showing AuNR precipitation. Therefore, the best combination of mPEG-SH and HS-PEG-COOH is in 9:1 ratio, so that more funtionalized AuNRs without aggregation for downstream cyto-*c* sensing could be obtained.

After the optimization of PEG ratio on AuNR conjugation, the quality of PEG-functionalized AuNRs on downstream functionalization with streptavidin and then the target aptamer was evaluated. Protein binding fluorescence SYPRO red dye was used to evaluate the amount of bound streptavidin protein, and DNA binding fluorescence SYBR safe dye was used to evaluate the amount of bound aptamer DNA. As expected, the previous optimized mPEG-SH:HS-PEG-COOH in a 9:1 ratio yielded the highest binding percentage of streptavidin and aptamer, compared with other ratios (Figure 3C,D). Theoretically, a higher binding percentage of streptavidin and aptamer should occur in a higher percentage of HS-PEG-COOH in PEG displacement, given that the free carboxyl group available is required for the EDC/NHS chemistry for conjugating the amino group in streptavidin. However, the high percentage of HS-PEG-COOH led to a higher loss of AuNR (Figure 3B). It is believed the positive charge on CTAB, similar to ionic salt, could attract the negatively-charged HS-PEG-COOH conjugated with AuNRs and lead to aggregation. However, CTAB cannot be fully removed prior to PEG displacement, as AuNRs will aggregate spontaneously without CTAB. The addition of mPEG-SH, a neutrally-charged PEG, could therefore reduce the aggregation during PEG conjugation, while a certain percentage of HS-PEG-COOH, in this case, a ratio of 9:1, retained the ability on AuNR functionalization with aptamer.

### 3.2. Signal Enhancement and Sensitivity Using the AuNR-Aptamer on AuNR-LSPR Assay

**Figure 4:**
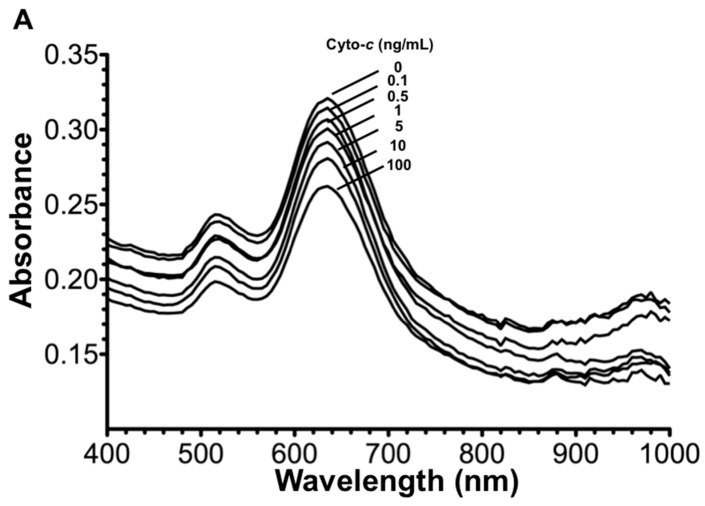

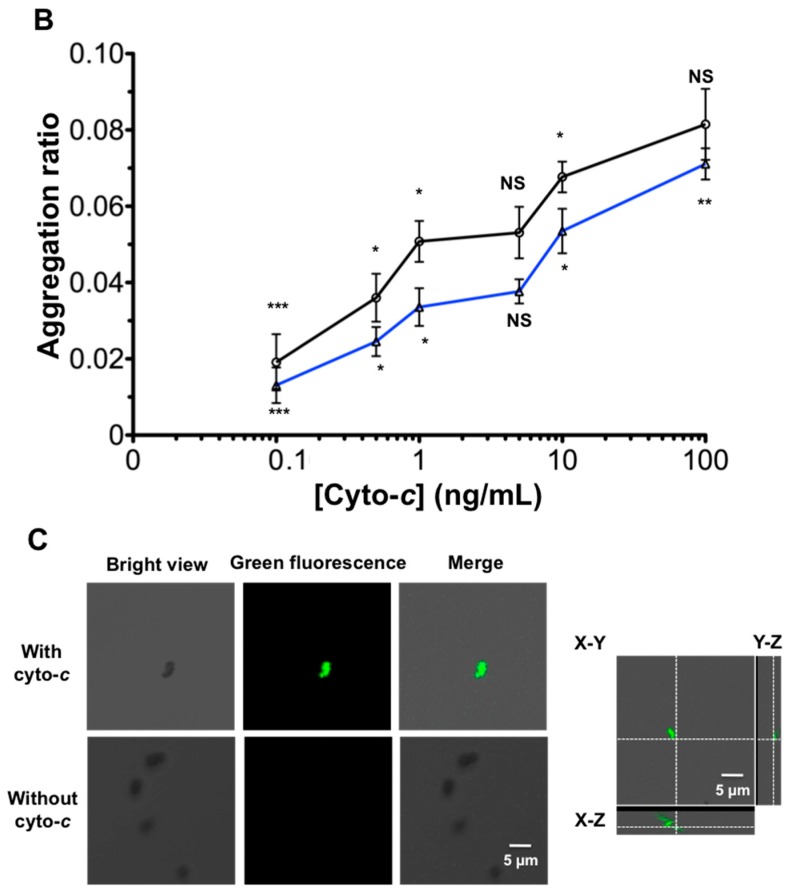
Sensitivity of cyto-*c* sensing. Cyto-c solutions of known concentrations (0, 0.1, 1, 10, 100 ng/mL) were used for AuNR-LSPR sensing. The absorbance spectrum (**A**) and the standard curve of the LSPR signal verse cyto-*c* concentrations were plotted (**B**). Results are mean ± SEM (*n* = 3), not significant (NS) *p* > 0.05, * *p* < 0.05, ** *p* < 0.01, *** *p* < 0.001. Confocal microscopy images showed the investigation of aptamer-functionalized green fluorescence AuNRs localizing and binding on MMP-Ab with or without cyto-*c*, where the right panel shows the 3D slicing of X-Y, Y-Z and X-Z axis (**C**).

After optimizing the AuNR-aptamer functionalization step, the AuNR-LSPR assay was performed with cyto-*c* standards of known concentration and the results were shown in Figure 4. As can be seen, aggregation of AuNR resulted in a redshift in the LSPR absorbance spectrum. To calculate the aggregation ratio, we used 760/630 for the AuNRs, where the numerator of 760 nm represented the uprising region of aggregated AuNRs, and the denominator of 630 nm represented the absorbance maximum or the peak region of the absorbance spectrum of non-aggregated AuNRs, respectively. The standard curve in Figure 4B showed the calculated aggregation ratio of standard cyto-*c* used in the AuNR-LSPR for the construction of a standard curve (Figure 4B).

The limit of detection (LOD) is defined by the International Union of Pure and Applied Chemistry (IUPAC) as the mean of the blank measured plus triple its standard deviation (SD) for a higher than 99% confidence level [27]. The SD of the aggregation ratio in the buffer control was found to be 0.006. Therefore, the LOD for cyto-*c* sensing using AuNR was calculated as follows: (0) + (3 × (0.006)) = 0.018 aggregation ratio unit for AuNR. The LOD of AuNRs was found below 0.1 ng/mL cyto-*c* from the standard curve. Standard concentration of cyto-*c* in culture medium was also used for a fair comparison of LOD with other studies that also used culture medium (Figure 4B). Although the overall detected signals were lower (blue curve), the LOD is similar to that in the serum (below 0.1 ng/mL cyto-*c)*, suggesting the culture medium did not pose any negative effects on sensitive detection in this assay.

In order to confirm whether aggregation has taken place from aptamer-functionalized AuNRs localizing and binding on the MMP-Ab-cyto-c complex rather than MMP-Ab without cyto-c, we conjugated AuNRs with green fluorescence protein (GFP) as a tag. DNA binding dye was avoided in this case since the binding action of the dye on DNA double strands would affect the secondary structure of aptamer DNA, thus altering its binding ability on cyto-*c*. It was found that green fluorescence only appeared in MMP complex when cyto-*c* was present (Figure 4C). To further evaluate how well the coverage of AuNRs on MMP-Ab-cyto-c was, *Z*-axis scanning with a resolution of 200 nm was conducted with confocal microscopy. The X-Y-Z graph showed the full coverage of MMP-Ab-cyto-*c* in the 2 μm-size dot, shown in the right panel of Figure 4C.

**Figure 5:**
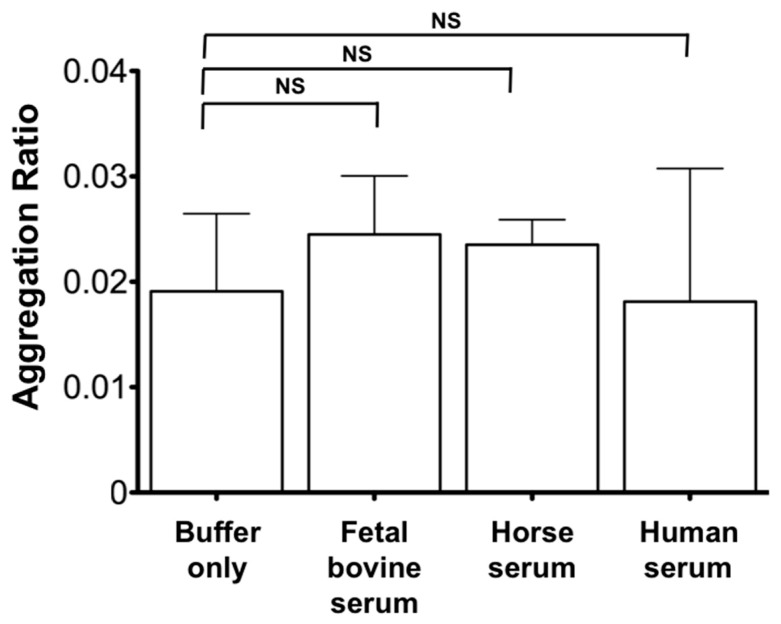
Utility of the AuNR-LSPR platform for cyto-*c* sensing in sera from different sources. Serum from fetal bovine, horse, and human were used to compare the utility of our AuNR-LSPR platform for cyto-*c* sensing. Cyto-*c* at a standard concentration of 0.1 ng/mL was sparked into these sera to evaluate the sensitivity of cyto-*c* sensing compared with that without serum. Results are mean ± SEM (n = 3), NS *p* > 0.05, * *p* < 0.05, ** *p* < 0.01, *** *p* < 0.001.

To sense the cyto-*c* in serum, we need to understand if any unwanted signals, which affect the LOD due to non-specific binding were found in a negative control in the serum. A standard solution of known cyto-*c* concentration in sera from different sources (e.g., fetal bovine, horse, and healthy human subjects) were prepared and used for cyto-*c* sensing. As expected, we did not see any significant difference of the cyto-*c* in sera when compared with that in buffer (Figure 5). In this context, we confirmed the utility of the AuNR-LSPR assay for cyto-*c* sensing in an environment with serum. Therefore, we can then proceed to the next step of the application.

### 3.3 Determination of Cyto-c Concentration in Serum Using AuNR-LSPR Assay 

**Figure 6:**
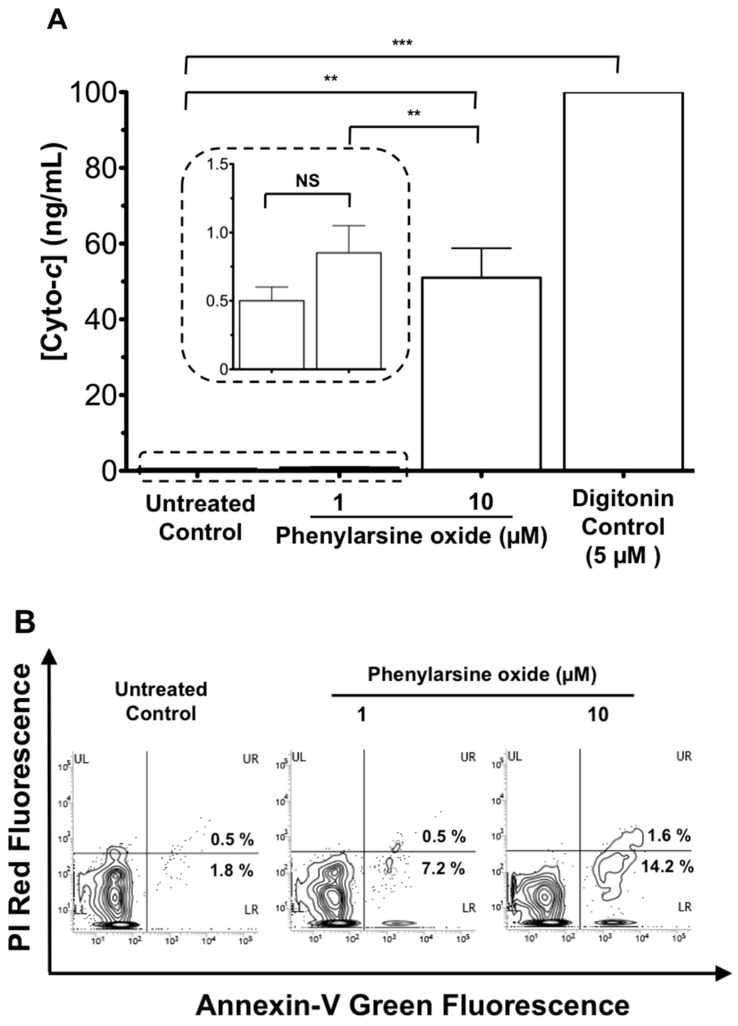
Cyto-*c* release during anti-cancer drug treatment using leukemia-sparked samples. HL-60 cells (1 × 10^5^/mL) in serum were cultured with the anti-cancer drug phenylarsine oxide at a concentration as indicated (1, 10 μM) or digitonin (5 μM) as a positive control at 37 °C, 5% CO_2_ for 1 h. (**A**) Cell-free sera were then collected by filtering with a 0.2 μm filter unit and subjected to the AuNR-LSPR assay for determining the release of cyto-*c*. Results are mean ± SEM (*n* = 3), NS *p* > 0.05, * *p* < 0.05, ** *p* < 0.01, *** *p* < 0.001. (**B**) Cells after treatment were labeled with Annexin-V-GFP and PI. After washing, cells were subjected to flow cytometric analysis for green and red fluorescence. The fluorescence properties of 1000 cells were collected for analysis.

Next, we tested the utility of our AuNR-LSPR assay with the cyto-*c* released from a leukemia cancer line HL-60 in serum after an in vitro anti-cancer drug treatment. In our study, PAO, a membrane-permeable tyrosine phosphatase inhibitor, was used as an anti-cancer agent and digitonin, a cell membrane detergent, was used as a positive control [15]. HL-60 cells (1 × 10^5^/mL) in serum were treated with serum alone, digitonin (5 μM), or PAO (1, 10 μM) for 1 h at 37 °C, and 5% CO_2_. Cell-free sera from the treated and control groups were collected by filtering them with a 0.2 μm filter unit, and were subjected to the AuNR-LSPR assay. As expected, a low concentration of cyto-*c* (0.5 ± 0.1 ng/mL) was found in the untreated control, indicating that cancer cells were intact in the serum. On the contrary, cyto-*c* was released upon PAO treatment in a dose-dependent manner (0.8 ± 0.2 ng/mL in 1 μM PAO; 40 ± 8 ng/mL in 10 μM PAO) and the digitonin-treated sample was found with a high concentration of cyto-*c* (>100 ng/mL). To verify the drug-induced apoptosis in the cells, Annexin-V-GFP, which targets the outer membrane leaflet during early apoptosis, and PI, which targets the nuclear DNA with a non-selective permeable or leaked plasma membrane during late apoptosis, were applied by cell staining for flow cytometric analysis. Obviously, we found only a small increase in green fluorescence in the lower right quadrant in the samples treated with PAO for one hour when compared with that of control (Figure 6B). This observation indicates that cyto-*c* release measured by our AuNR-LSPR assay was much more sensitive than flow cytometric Annexin-V and PI assays when the incubation time with the anti-cancer agent was short.

Indeed, we have developed a number of platforms using aptamers to determine the cyto-*c* concentration with different sensing techniques in the past few years. In Table 1, we compared the hardware requirement, signal amplification of different techniques, and their LOD on cyto-*c* sensing. Among all the techniques, our aptamer-based LSPR assay shares the advantage with the aptamer-based enzyme-linked immunosorbent assay (ELISA) method that a simple absorbance reader is required to carry out signal measurement. Additionally, because of the strong LSPR effect in the AuNR-SPR assay, the detection limit on cyto-*c* reported in this study was better than the other techniques using enzymes for signal amplification.

There are reports showing the change in serum cyto-*c* concentration in patients with different diseases. For example, the serum concentration of cyto-*c* in patients with SIRS was increased up to 210 ng/mL compared with that of healthy subjects (<0.1 ng/mL) [9,10]. In the patients with benign tumors, the serum cyto-*c* concentration was below 20 ng/mL, while that in the patients with malignant tumors and low survival rate (over 75% of the patients) was found to be over 40 ng/mL [28]. Obviously, our AuNR-LSPR platform is able to cover this range of serum cyto-*c* concentration in the above scenarios for clinical diagnosis. Additionally, the simple operation using LSPR absorbance change enables health providers, who are not trained for the medical laboratory techniques, to read the results easily. In fact, this AuNR-LSPR assay using the microfluidic format could extend to test other disease markers (e.g., tumor-specific markers) easily with a simple replacement of the corresponding recognition elements. We expect that this detection platform will have a variety of uses in the coming future.

## 4. Conclusions

We have demonstrated a new approach using a label-free LSPR format with AuNRs for cyto-*c* sensing in human serum to monitor the efficacy of cancer treatment. This AuNR-LSPR platform is rapid, robust, and simple. In the presence of cyto-*c*, the AuNRs conjugated with cyto-*c*-specific aptamers will form aggregates, thus giving rise the LSPR effects. Practically, the LOD for cyto-*c* was found to be 0.1 ng/mL in the buffer, culture medium, and serum of different animals, including fetal bovine, horse, and human. As a proof of concept, we have successfully demonstrated the utility of this platform to detect the cyto-*c* released from leukemia cells upon anti-cancer drug PAO treatment. Taken together, our simple AuNR-LSPR detection platform is expected to have a wide range of potential for detecting disease markers in the future.

## Figures and Tables

**Table 1:** Comparison of cyto-*c* sensing using aptamer via different techniques.

Technique	Hardware Requirement	Signal Amplification	Detection Limit (ng/mL)	Reference
Aptamer-based LSPR	Absorbance reader	–	0.1	This study
Aptamer-based SPR	SPR sensor	–	1 (80 pM)	[12]
ABC-Rash assay-RNase H digestion on RNA probes	SPR sensor	Enzyme-based	1 (80 pM)	[15]
ABC-PCR assay	Real-time fluorescence thermocycler	Enzyme-based	10	[14]
ABC-RPA (Recombinase polymerase amplification) assay	Real-time fluorescence detector with temperature regulation	Enzyme-based	10	[13]
Aptamer-base ELISA	Absorbance reader	Enzyme-based	100	[12]
